# Characterization of systolic and diastolic function, alongside proteomic profiling, in doxorubicin-induced cardiovascular toxicity in mice

**DOI:** 10.1186/s40959-024-00241-1

**Published:** 2024-06-22

**Authors:** Dustin N. Krüger, Matthias Bosman, Charles X.L. Van Assche, Callan D. Wesley, Berta Cillero-Pastor, Leen Delrue, Ward Heggermont, Jozef Bartunek, Guido R. Y. De Meyer, Emeline M. Van Craenenbroeck, Pieter-Jan Guns, Constantijn Franssen

**Affiliations:** 1 Laboratory of Psychopharmacology, Faculty of Medicine and Health Sciences, Faculty of Pharmaceutical, Biomedical and Veterinary Sciences, Campus Drie Eiken, University of Antwerp, Universiteitsplein 1, Antwerp, B-2610 Belgium; 2 Division M4I – Imaging Mass Spectrometry (IMS), Faculty of Health, Medicine and Life Sciences, Maastricht MultiModal Molecular Imaging Institute, Maastricht University, Universiteitssingel 50, Maastricht, 6229 ER The Netherlands; 3 Research Group Cardiovascular Diseases, University of Antwerp, Wilrijkstraat 10, Edegem, B-2650 Belgium; 4 Department of Cardiology, Antwerp University Hospital (UZA), Drie Eikenstraat 655, Edegem, B-2650 Belgium; 5 Department of Cell Biology-Inspired Tissue Engineering, Institute for Technology-Inspired Regenerative Medicine, Universiteitssingel 40, Maastricht, 6229 ER The Netherlands; 6 Cardiovascular Centre, OLV Hospital, Moorselbaan 164, Aalst, B-9300 Belgium

**Keywords:** Doxorubicin, Cardiovascular toxicity, Serpina3, Diastolic dysfunction, Heart failure, Biomarker

## Abstract

**Background:**

The anthracycline doxorubicin (DOX) is a highly effective anticancer agent, especially in breast cancer and lymphoma. However, DOX can cause cancer therapy-related cardiovascular toxicity (CTR-CVT) in patients during treatment and in survivors. Current diagnostic criteria for CTR-CVT focus mainly on left ventricular systolic dysfunction, but a certain level of damage is required before it can be detected. As diastolic dysfunction often precedes systolic dysfunction, the current study aimed to identify functional and molecular markers of DOX-induced CTR-CVT with a focus on diastolic dysfunction.

**Methods:**

Male C57BL/6J mice were treated with saline or DOX (4 mg/kg, weekly i.p. injection) for 2 and 6 weeks (respectively cumulative dose of 8 and 24 mg/kg) (*n* = 8 per group at each time point). Cardiovascular function was longitudinally investigated using echocardiography and invasive left ventricular pressure measurements. Subsequently, at both timepoints, myocardial tissue was obtained for proteomics (liquid-chromatography with mass-spectrometry). A cohort of patients with CTR-CVT was used to complement the pre-clinical findings.

**Results:**

DOX-induced a reduction in left ventricular ejection fraction from 72 ± 2% to 55 ± 1% after 2 weeks (cumulative 8 mg/kg DOX). Diastolic dysfunction was demonstrated as prolonged relaxation (increased tau) and heart failure was evident from pulmonary edema after 6 weeks (cumulative 24 mg/kg DOX). Myocardial proteomic analysis revealed an increased expression of 12 proteins at week 6, with notable upregulation of SERPINA3N in the DOX-treated animals. The human ortholog SERPINA3 has previously been suggested as a marker in CTR-CVT. Upregulation of SERPINA3N was confirmed by western blot, immunohistochemistry, and qPCR in murine hearts. Thereby, SERPINA3N was most abundant in the endothelial cells. In patients, circulating SERPINA3 was increased in plasma of CTR-CVT patients but not in cardiac biopsies.

**Conclusion:**

We showed that mice develop heart failure with impaired systolic and diastolic function as result of DOX treatment. Additionally, we could identify increased SERPINA3 levels in the mice as well as patients with DOX-induced CVT and demonstrated expression of SERPINA3 in the heart itself, suggesting that SERPINA3 could serve as a novel biomarker.

**Supplementary Information:**

The online version contains supplementary material available at 10.1186/s40959-024-00241-1.

## Background

Doxorubicin (DOX) is an anthracycline chemotherapeutic and represents a cornerstone in the treatment of many malignancies, especially breast cancer and lymphoma. However, DOX is also associated with potential cancer therapy-related cardiovascular toxicity (CTR-CVT). CTR-CVT is defined as a significant worsening of cardiac function during and after cancer treatment [1]. It can be classified from mild toxicity in asymptomatic patients (with higher levels of circulating biomarkers troponin and natriuretic peptide) to severe toxicity, characterized by overt left ventricular (LV) dysfunction and heart failure (HF). CTR-CVT leads to increased morbidity, mortality and reduced quality of life in the growing population of cancer survivors [1, 2]. One important risk factor of DOX-induced CVT among common CV risk factors is cumulative dosing [3]. It was shown that the risk of HF is 5% in patients with a cumulative dose of 400 mg/kg^2^, while it is above 45% when patients receive a cumulative dose of ≥ 700 mg/m^2^ (reviewed by [4]). Despite variations in reported incidence due to differing definitions of CVT, it is generally agreed that cumulative doses of ≥ 250 mg/m^2^ are already associated with risk of CTR-CVT [1].

Current guidelines on cardio-oncology advise screening for CTR-CVT with cardiac-specific circulating biomarkers (i.e., troponin and natriuretic peptides) and cardiac imaging (echocardiography) with a particular focus on left ventricular ejection fraction (LVEF) and global longitudinal strain (GLS) imaging [1]. However, identifying subtle alterations in LV function can pose challenges. Conversely, circulating biomarkers, which can be influenced by factors such as age, sex, and comorbidities [5–8], are frequently inconsistent and thus not commonly employed in clinical settings. Moreover, current plasma markers insufficiently reflect the pathophysiology of CTR-CVT [1].

Several reports have proposed endothelial dysfunction as a potential early marker of DOX-induced cardiotoxicity [9]. Recently, we documented vascular toxicity, represented by endothelial cell (EC) dysfunction and arterial stiffness, as a predecessor to the reduced LVEF upon DOX treatment in mice [10, 11]. Thereby, EC dysfunction was reversible after stopping DOX treatment [12]. Endothelial dysfunction contributes to vascular contractile alterations and reduced compliance in both heart and aorta, compromising cardiac relaxation and filling, which can ultimately lead to diastolic dysfunction, or heart failure with preserved ejection fraction (HFpEF) [13, 14]. Functional assessment in patients revealed that early signs of DOX-induced CTR-CVT may present as diastolic dysfunction [15], which could precede systolic impairment or HF with reduced LVEF (HFrEF) [16].

In the current study, we aimed to identify early cardiac functional markers for DOX-induced CVT in mice with a focus on diastolic (dys)function. Additionally, we explored changes in protein expression by proteomics to identify novel molecular biomarkers and validated them in patients.

## Methods

### Animals

For all experiments, male C57BL/6J mice (Charles River, France) at the age of 10 weeks and weighing between 25 and 30 g were studied. The mice were housed in standard cages in the animal facility at the University of Antwerp, with access to regular chow and water ad libitum, with 12–12 h light-dark cycles.

### Study design

Mice were randomly divided into vehicle, (*n* = 16) or treatment (*n* = 16) groups. Treated animals were injected intraperitoneally with 4 mg DOX/kg (Adriamycin^®^, 2 mg/mL, Pfizer, Belgium) once per week, controls with 0.9% NaCl solution (10 mL/kg, B. Braun, Belgium), once per week. Mice underwent longitudinal follow-up by echocardiography at baseline (week 0), week 2, and week 6. Animals were sacrificed after 2 and 6 weeks of DOX treatment (respectively cumulative dose of 8 and 24 mg/kg). At the end of the study, LV function was assessed terminally by inserting a pressure catheter in the LV. Afterwards, the animals were killed by an overdose of anaesthetics (sodium pentobarbital, 200 mg/kg. i.p.) and organs were collected and weighed for further analysis. For proteomics, murine cardiac tissue from a historical study was used, previously reported in Bosman et al. [12]. These mice underwent the same treatment protocol as the animals used in this study.

### Ultrasound imaging

Mice were first anaesthetized using isoflurane (induction concentration of 3% v/v) and were maintained sedated at 1–2% v/v. They were then placed on the Vevo F2 LAZR-X imaging station (Visual Sonics - FUJIFILM), which is heated to stabilize the body temperature of the animals at 37 ± 1 °C and enabled the recording of physiological parameters. Heart rate and respiration were constantly monitored and maintained at approximately 400–500 beats per minute and 100 breaths per minute, respectively.

Echocardiographic measurements were carried out using a 57 MHz transducer (Visual Sonics). Cardiac images were recorded from different perspectives, including parasternal long-axis and short-axis views, as well as the apical four-chamber view. LVEF and fractional shortening (FS), were measured as indices of systolic function (M-mode). Additionally, diastolic function was assessed using the mitral valve E/A ratio (MV E/A) and the E/E’ ratio (MV E/E’). These measurements were performed on at least three heartbeats from each view and then averaged.

### Left ventricular pressure analysis

At the end of experiment left ventricular pressure (LVP) analysis was conducted as terminal endpoint, to investigate in depth contractile function of LV. Mice were anaesthetized using sevoflurane (8% v/v for induction and 4–5% v/v for maintenance). To maintain body temperature at approximately 37 °C, the animals were placed on a heating pad (Kent Scientific). Surgical preparation involved isolation of the right carotid artery for catheter insertion. For left ventricular catheterization, a pressure catheter (Millar SPR-671) was inserted into the LV through the right carotid artery using a closed-chest approach.

### Proteomics

Label-free proteomics was used to analyze protein expression changes induced by DOX treatment from fresh frozen cardiac tissue (apex). Protein isolates were digested to peptides with trypsin before use. These samples were desalted on C18 trapping column. After desalting peptides were separated on the PepSep C18 analytical column (15 cm, ID 75 µm, 1.9 µm Reprosil, 120 Å). The Ultimate 3000 Rapid Separation ultra-high performance liquid chromatography (UHPLC) system was coupled to a Q Exactive HF mass spectrometer (Thermo Scientific) with the following settings: Full MS scan between 250–1,250 m/z at a resolution of 120,000 followed by MS/MS scans of the top 15 most intense ions at a resolution of 15,000. A detailed description of the method is published elsewhere [12].

Finally, functional enrichment analysis on the differentially expressed proteins was performed using Enrichr [17, 18], with the Reactome 2022 Human database. Enrichment was performed for up- and downregulated proteins separately, as this provides a more accurate and powerful analysis [19].

### Western blot

For western blot, the same protein extract samples of cardiac apex as for proteomics were used. Before electrophoresis, the protein solution was reduced in Laemmli buffer (Bio-Rad, Temse, Belgium) with 5% β-mercaptoethanol (Sigma-Aldrich, Overijse, Belgium) and denatured for 5 min at 100 °C. Afterwards, 10–20 µg of proteins were loaded per band on a 4–12% Bis-Tris gel (Invitrogen, Merelbeke, Belgium). The gel was transferred to Immobilon-FL PVDF membranes (Merck, Hoeilaart, Belgium). After blocking (Odyssey Li−COR blocking buffer (Li-COR Biosciences, Bad Homburg, Germany)), membranes were probed with primary antibodies anti-SERPINA3N (1:1000; AF4709, R&D systems, UK), and mouse anti-GAPDH (1:5000; ab8226, Abcam, Cambridge, UK), diluted in Odyssey Li−COR blocking buffer, overnight at 4 °C. The next day, membranes were incubated with IRDye-labeled secondary antibodies (goat anti-rabbit IgG926−32211; goat anti-mouse IgG926−68070, (Li−COR Biosciences, Bad Homburg, Germany) for 1 h at room temperature. Membranes were visualised with an Odyssey SA infrared imaging system (Li−COR Biosciences Bad Homburg, Germany). Western blot data were quantified using ImageJ software. Signal intensity of SERPINA3N was normalised to the GAPDH and expressed as fold change (compared to vehicle).

### Histology

After dissection, cardiac and thoracic aortic tissues were washed in Krebs-Ringer solution, immediately submerged in 4% formaldehyde, buffered pH = 7 (Merck, Overijse, Belgium) and stored at 4 °C for 24 h. The following day segments were placed in 30% sucrose for an additional 24 h at 4 °C. Afterwards, the tissues were embedded in frozen section medium NEG-50™ (Epredia, Belgium) and frozen at -21 °C. On the day of staining for immunohistochemistry (IHC), segments were transversely cut into 10 µm segments. A primary antibody against SERPINA3N (1:500; AF4709 R&D Systems, UK) was used to investigate SERPINA3N distribution in cardiac tissue. Followed by visualization using a rabbit anti-goat biotinylated secondary antibody (1:200; Vector Laboratories, Burlingame, USA), cardiac tissues were stained with aminoethyl carbazole (Sigma Aldrich, Overijse, Belgium). The nuclei of the cells were stained at the end with hematoxylin. Sections for hematoxylin and eosin (H&E) as well as for Masson’s Trichome staining, were fixated in paraffin. Both stains were performed in the Epredia™ Gemini AS staining machine (Thermo Fisher, Belgium).

### Cardiac mRNA expression (qPCR)

Total RNA was extracted from the heart apex using the ISOLATE II RNA Mini Kit (Bioline Meridian, Netherlands) following the given protocol. Tissue was completely homogenized using a Mini-Beadbeater (2 times 20 s at 5000 rpm). Purity and concentration of the RNA were measured using a spectrophotometer ND-1000 (NanoDrop®) and stored afterwards at -80 °C. 300–500 ng of RNA were transcribed into cDNA by making use of the TaqMan™ Reverse Transcription Reagents kit (Invitrogen). Random hexamers were used as primers (2.5 µM) included in the manufactures kit. Quantitative polymerase chain reaction (qPCR) using the TaqMan Fast Advanced Master Mix Kit (Applied Biosystems™) was performed to determine myocardial mRNA expression of *Il-1β*, *Il-6*, *Tnfα, Serrpina3n* and *Gapdh* using TaqMan probes (Thermo Fisher Scientific, Mm00434228_m1, Mm00446190_m1, Mm00443258_m1, Mm00776439_m1 and Mm99999915_g1).

### Circulating SERPINA3 in mice (ELISA)

Plasma SERPINA3 levels were analyzed using the SERPINA3N mouse ELISA kit OKEH03592 (Aviva Systems Biology, California, USA). Plasma samples were diluted 1:10 in dilution buffer of the kit. The kit was performed according to the manufacturer’s instructions. Afterwards, absorbance was measured with a plate reader at 450 nm.

### Analysis of SERPINA3 in patients with CTR-CVT

Two groups of human samples were included in the study: cancer survivors experiencing CTR-CVT (*n* = 24), and a control group of patients without prior cancer treatment, with preserved LVEF, undergoing cardiac catheterization for non-HF symptoms (*n* = 5). Of note, this cohort differs from the one previously described [12]. Blood samples were taken at diagnosis of CTR-CVT (77 ± 3 months after completion of cancer treatment) or at diagnosis of cardiomyopathy in the control cohort. LV biopsies were procured from all patients during catheterization and *SERPINA3* expression was determined. Circulating SERPINA3 levels were measured using commercially available ELISA kits (Human Alpha 1-Antichymotrypsin ELISA, Immunology Consultants Laboratory, Inc., USA, by Bio-Techne Ltd®, R&D Systems®, Abingdon, UK) following the manufacturer’s instructions. Myocardial expression of *SERPINA3* was measured by qPCR. RNeasy Fibrous Tissue Mini Kit (Qiagen Venlo, Netherlands) was used for RNA extraction. qPCR was subsequently performed with TaqMan Gene Expression Assays using a 7500 real‐time PCR system (Applied Biosystems). Expression of *SERPINA3* (Hs00153674_m1) was normalized to *GAPDH* (Hs99999905_m1) in the same sample.

### Data analysis

For analysis of all data Prism 9.0 (GraphPad Software, La Jolla, CA, USA) was used. Results are expressed as mean ± SEM, with *n* representing the number of mice. For the echocardiographic data, between-group differences were assessed by a two-way ANOVA (mixed effects) and a Geisser-Greenhouse correction. Weight, western blots, ELISA and qPCR results were analyzed with the Mann-Whitney test for each time point. Statistical significance was defined as *p* ≤ 0.05.

Proteomics were analyzed using the Protein Discoverer software (version 2.2), while search engine Sequest was used with SwissProt mouse protein database (Mus Musculus, SwissProt TaxID = 10090). Correlation analysis of patient-related data and their SERPINA3 expression in plasma and myocardial tissue was performed using IBM SPSS Statistics (Version 29.0.2.0).

## Results

### Evaluation of DOX-induced cardiotoxicity in mice

**Fig. 1:**
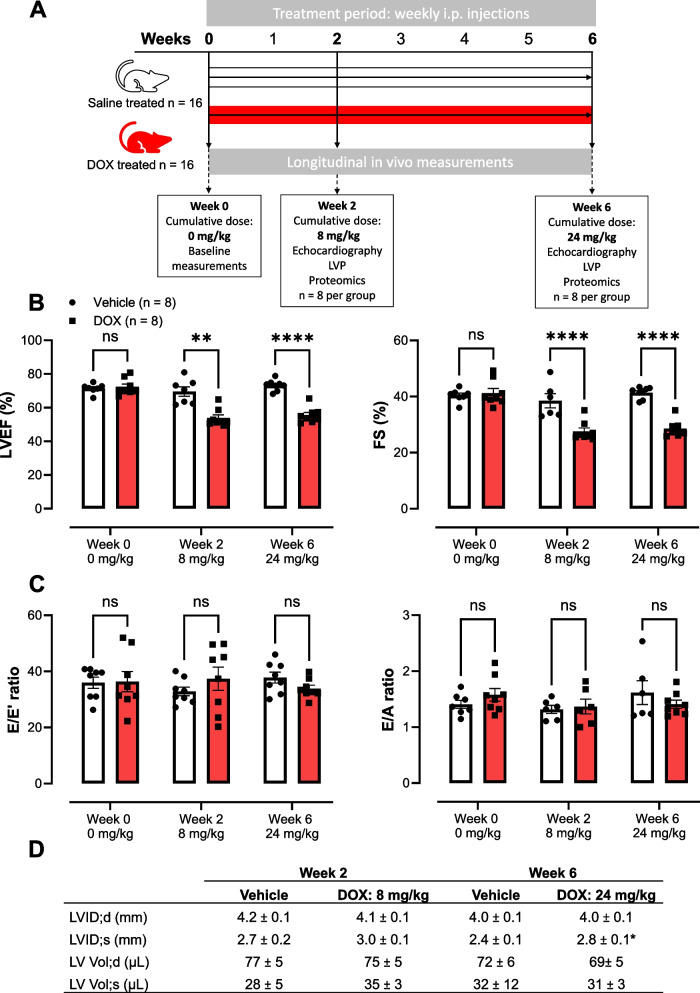
Cardiac function during DOX treatment, measured by echocardiography over a period of 6 weeks. **A** Experimental protocol. **B** During DOX treatment, LVEF and FS decreased from week 2. **C** No changes in diastolic function were detected. **D** LV parameters measured by ultrasound imaging. LVID;d, Left ventricular inner diameter during diastole. LVID;s, Left ventricular inner diameter during systole. LV Vol;d, Left ventricular volume, diastole. LV Vol;s, Left ventricular volume, systole. LVP, Left ventricular pressure. LVEF, Left ventricular ejection fraction. FS, fractional shortening. E, peak velocity blood flow in early diastole. A peak velocity blood flow in late diastole. E’, tissue Doppler-derived mitral valve velocity in early diastole. Doses represent cumulative dose of DOX. Data shows mean ± SEM. **B** & **C** were analyzed using a Two-Way ANOVA (mixed effects) and a Geisser-Greenhouse correction. For each cohort: *n* = 8 in both groups. **, ****, *p* < 0.01, 0.0001. **D** Mann-Whitney test between DOX and vehicle for each time point. * *p* ≤ 0.05

A reduction in LVEF was set as confirmation of CTR-CVT. A detailed echocardiographic analysis of the DOX murine model has already been performed by Bosman et al., focusing on vascular and LV systolic function. We previously described EC dysfunction occurring early on in DOX-induced CTR-CVT [12]. The present study focuses on diastolic dysfunction and the identification of molecular changes in DOX-induced CTR-CVT in the aforementioned model. The mice received weekly injections of either DOX (4 mg/kg) or Saline. Cardiac function was measured at baseline (week 0) or at a cumulative dose of 8 mg/kg (week 2), or 24 mg/kg (week 6). While some of the mice were treated weekly with DOX for up to 6 weeks, resulting in a cumulative dose of 24 mg/kg, no mortality was observed. A detailed experimental plan, including the treatment protocol, is shown in Fig. 1A.

**Fig. 2:**
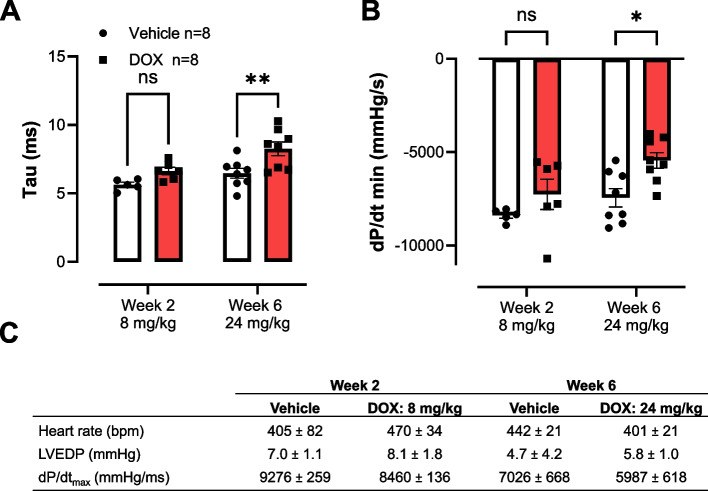
Invasively measured LV hemodynamic parameters. **A** Increased relaxation time constant Tau after 6 weeks. **B** Reduction in dP/dt_min_ after 6 but not 2 weeks. **C** No changes in LVEDP, heart rate and dP/dt_max_. Doses represent cumulative dose of DOX. Values are in mean ± SEM. *n* = 8. LVEDP, left ventricular end-diastolic pressure. **A** & **B** Mixed-effects analysis ** *p* < 0.001, * *p* < 0.05. **C** Mann-Whitney test between DOX and vehicle for each time point

In line with our previous findings, DOX reduced LVEF (72 ± 2% to 55 ± 1%) and FS (41 ± 2% to 28 ± 1%) after 2 weeks (respectively cumulative dose of 8 mg/kg) consistent until the mice reached the final cumulative dose of 24 mg/kg (week 6) (Fig. 1B). However, LVEF did not decrease below 50%. Apart from changes in systolic function, DOX did not lead to measurable diastolic dysfunction on cardiac ultrasound, as evident from similar E/A and E/E´ ratios compared to controls (Fig. 1C). In contrast, when measured invasively, DOX-induced diastolic impairment. Specifically, invasive LV pressure analysis showed no alterations in systolic function after DOX treatment (dP/dt_max_, Fig. 2C). Relaxation phase was altered; the time constant for isovolumetric LV pressure decline Tau (τ) was prolonged after 6 weeks of DOX (8.3 ± 0.5 ms) compared to the controls (6.5 ± 0.4 ms), indicative of diastolic dysfunction (Fig. 2A). Furthermore, dP/dt_min_ was reduced from -7727 ± 463 mmHg/s to -5816 ± 436 mmHg/s after 6 weeks (Fig. 2B). LV end-diastolic pressure (LVEDP) was not altered after DOX treatment.

**Fig. 3:**
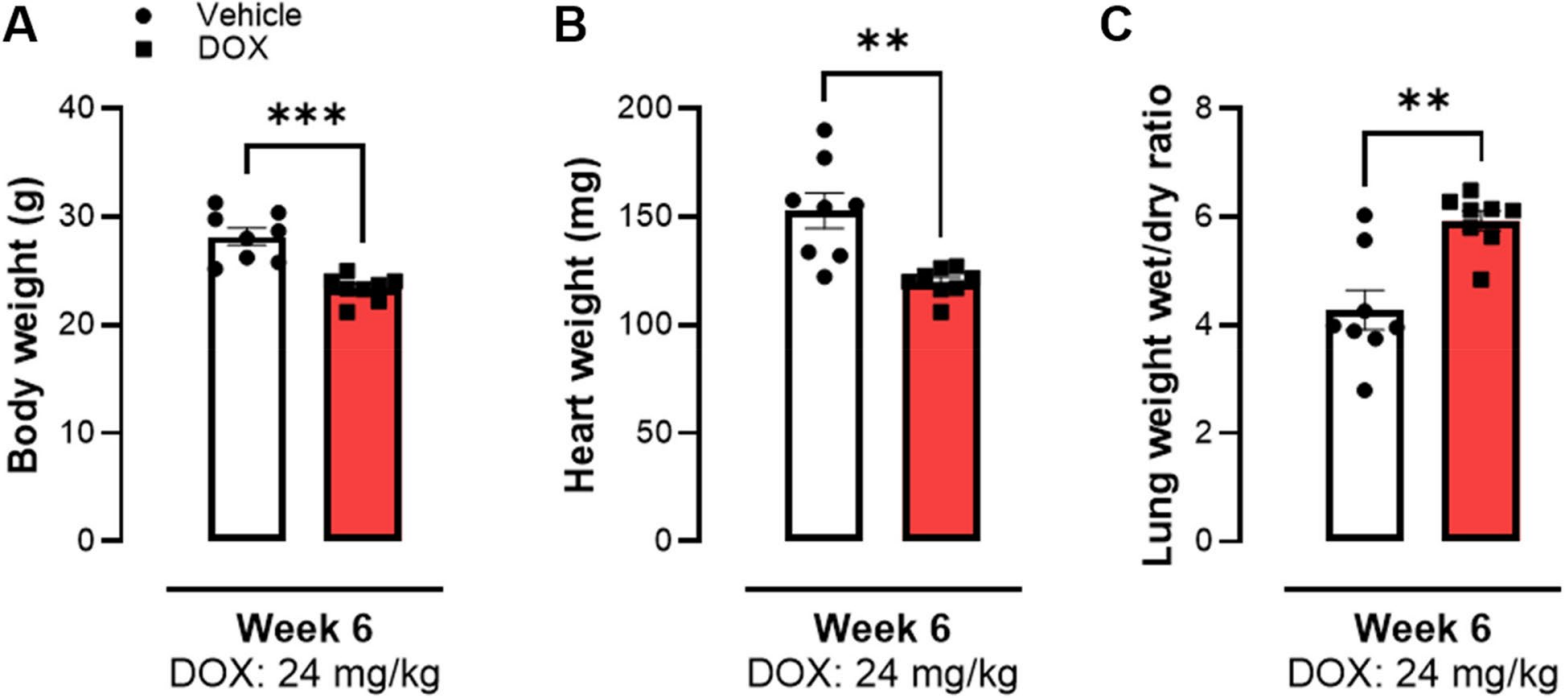
Body- and organ-weight assessment from mice after 6 weeks of DOX treatment. Mice showed a reduction in body weight as well as in heart weight. **A** Reduced body weight in treated group. **B** Heart weight was decreased after DOX treatment. **C** Lung weight wet/dry ratio was significantly increased in the treated group. Doses represent cumulative dose of DOX. Data shows mean ± SEM. **A** - **C** Mann-Whitney test between DOX and vehicle group. **, *** *p* < 0.01, 0.005

After 6 weeks of DOX treatment, mice exhibited a reduction in body weight compared to the control group, as well as reduced heart weight (Fig. 3A & B). Additionally, lung weight (wet-to-dry ratio) was increased in DOX-treated animals (Fig. 3C).

**Fig. 4:**
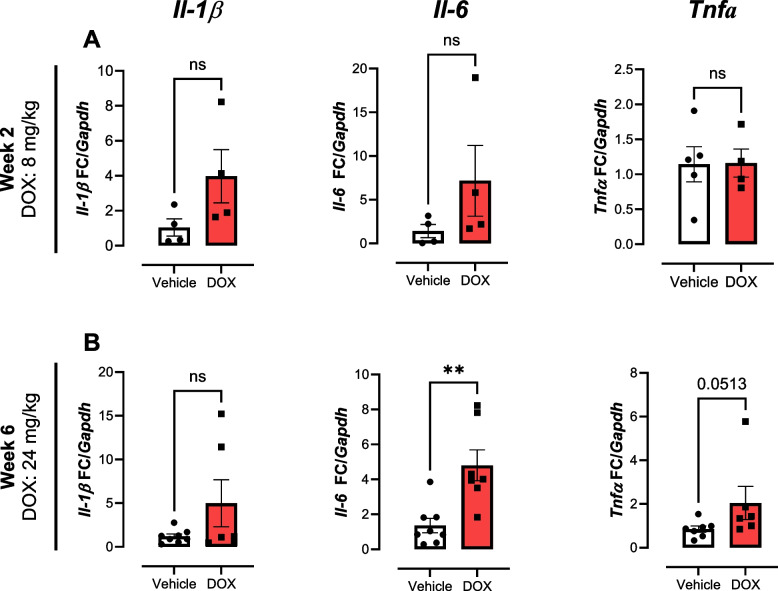
Analysis of Inflammatory markers in cardiac tissue by qPCR. **A** DOX treatment for 2 weeks did not alter gene expression of *Il-1β*, *Il-6* and *Tnfα* in the treated mouse cardiac tissue. **B** After 6 weeks of treatment *Il-6* expression was increased. FC, Fold change. Doses of DOX represent cumulative dose. Data shows mean ± SEM. At 2 weeks, *n* = 5 for vehicle and *n* = 4 for DOX. Week 6 *n* = 8 for vehicle and *n* = 7 for DOX. For **A** & **B** Mann-Whitney test for each time point. ** *p* < 0.01

DOX is known to induce systemic inflammation [20]. Therefore, we investigated mRNA content of common cytokines *IL-1β*, *Il-6* and *Tnfα* in cardiac tissue at both time points. After 2 weeks (cumulative dose of 8 mg/kg of DOX), *Il-1β* and *Il-6* tended to slightly increase, although not reaching significance (Fig. 4A). After 6 weeks of DOX administration, *Il-6* mRNA content was increased (~5-fold), whereas no effects were detected on *Il-1β* (Fig. 4B). Noteworthy, a trend towards increased *Tnfα* gene expression was observed, but did not reach statistical significance (*p* = 0.0513).

**Fig. 5:**
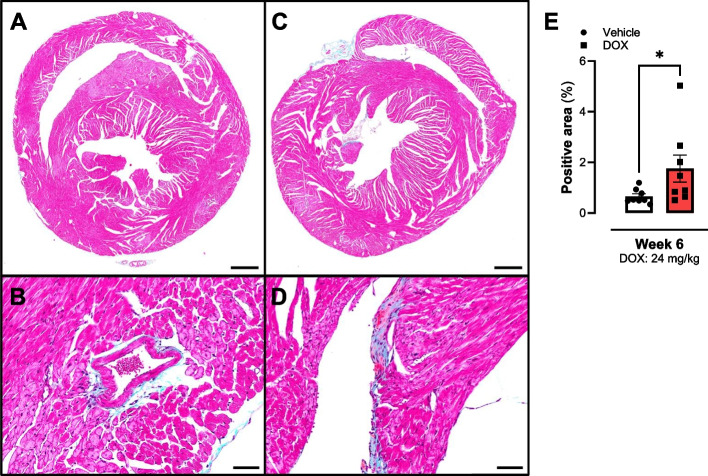
Fibrosis analysis of cardiac tissue from mice at 6 weeks (24 mg/kg). DOX treatment increased the positive Masson’s Trichome area in murine cardiac tissue. **A** & **B** Representative image of cardiac tissue from a vehicle-treated mouse. **C** & **D** Representative image of cardiac tissue from a DOX-treated mouse. Dose represents cumulative dose of DOX. Data shows mean ± SEM. **A** & **C** Scale bar 500 µm. **B** & **C** Scale bar 50 µm. **E** Mann-Whitney test between Masson’s Trichome positive areas from whole cardiac sections. * *p* < 0.05

DOX-treated mice showed more fibrotic area on a Masson’s Trichome staining at 6 weeks as shown in Fig. 5E. Figure 5 includes representative images of cardiac tissue from vehicle (Fig. 5A & B) and DOX-treated (Fig. 5C & D) animals. Furthermore, no histopathology alterations were detected on H&E at week 6 (Supplementary Figure 1).

### Identification of myocardial molecular changes by proteomics

**Fig. 6:**
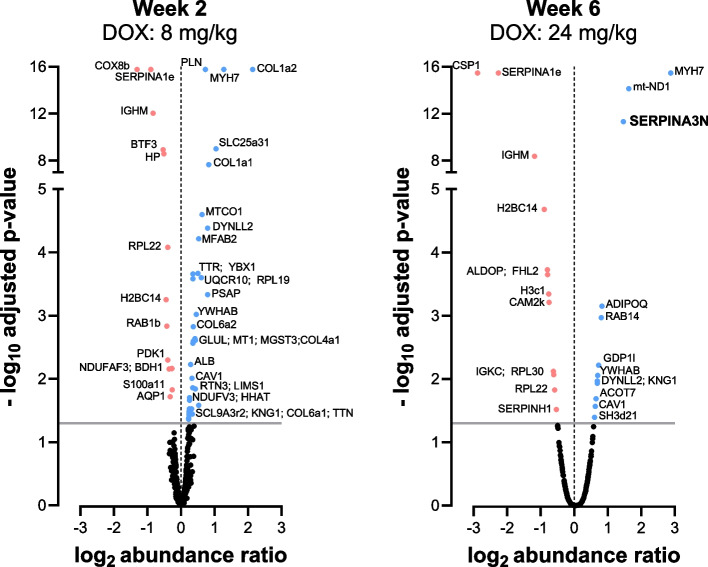
Proteomics of cardiac tissue from DOX-treated mice at 2 and 6 weeks. The significance threshold (*p* < 0.05) is represented by a horizontal grey line. Upregulated proteins are highlighted in blue, while downregulated proteins are marked in red. The proteins are labelled with the corresponding gene name

To further investigate possible molecular markers of DOX-induced cardiotoxicity, proteomic analysis was performed on cardiac tissue after different cumulative doses of DOX. 1019 proteins with high confidence were identified. After 2 weeks of DOX treatment (cumulative 8 mg/kg DOX), 41 proteins were up- and 13 down-regulated whereas only 12 proteins were up- and 12 were down-regulated after 6 weeks of DOX treatment (cumulative 24 mg/kg). A summary of proteins with altered expression is shown as volcano plots in Fig. 6. A more detailed list of the differentially up- and down-regulated proteins and their corresponding names is provided in a Supplementary Excel^®^ file. At 2 and 6 weeks, MYH7 was increased and SERPINA1E was decreased. Pathway analysis was performed using *Enrichr* to identify pathways, which could potentially be involved in disease mechanisms (Supplementary Figure 2). Additionally, literature research was conducted on most up-and-down regulated proteins to identify new possible candidates as markers for CTR-CVT. Based on literature we chose SERPINA3N (hereafter called SERPINA3, also known as α-1-antichymotrypsin) for further validation.

#### Myocardial SERPINA3

**Fig. 7:**
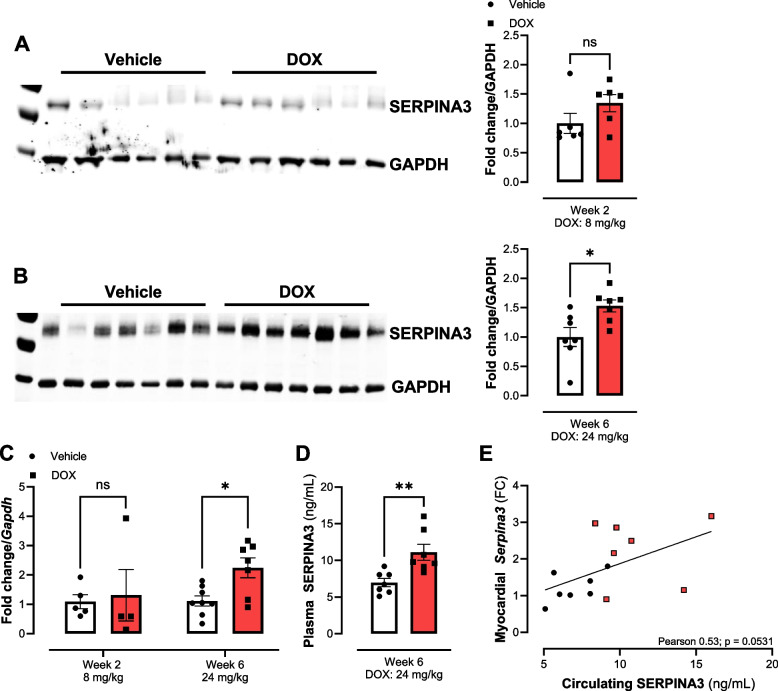
Validation of SERPINA3 protein content and gene expression in murine cardiac tissue at week 2 and 6. **A** & **B** Western blots and quantification for validation of proteomics findings of increased SERPINA3 levels after 6 weeks of treatment. Protein concentration did not change at 2 weeks. **C** mRNA level of Serpina3 (qPCR) at 6 weeks was increased, whereas after 2 weeks no change was observed. **D** Circulating SERPINA3 in plasma of mice treated for 6 weeks was increased. **E** Correlation between myocardial expression and circulating SERPINA3 at cumulative dose of 24 mg/kg DOX. FC, Fold change. Data shows mean ± SEM. **A** *n* = 6 for DOX and vehicle. **B** & **D** *n* = 7 per group. **C** At 2 weeks, *n* = 5 for vehicle and *n* = 4 for DOX. Week 6 *n* = 8 for vehicle and *n* = 7 for DOX. For **A** - **D** Mann-Whitney test for each time point. *, ** *p* < 0.05, *p* < 0.01. **E** Pearson correlation

To confirm differential expression of SERPINA3, western blot and qPCR were conducted. Protein content of SERPINA3 (Fig. 7, fold change to GAPDH) increased at 6 weeks, which is in line with findings from proteomic analysis (Fig. 7B). At week 2, protein levels of SERPINA3 were not significantly altered. Uncropped blots are shown in Supplementary Figure 3. Total mRNA content of *Serpina3* was not changed at week 2 but increased at week 6 (Fig. 7C). Circulating SERPINA3 was increased after 6 weeks of DOX treatment compared to vehicle-treated mice (Fig. 7D). Myocardial mRNA expression tended (*p* = 0.0531) to correlate with circulating SERPINA3 in plasma of mice.

### Localization of SERPINA3 in myocardial tissue of mice

**Fig. 8:**
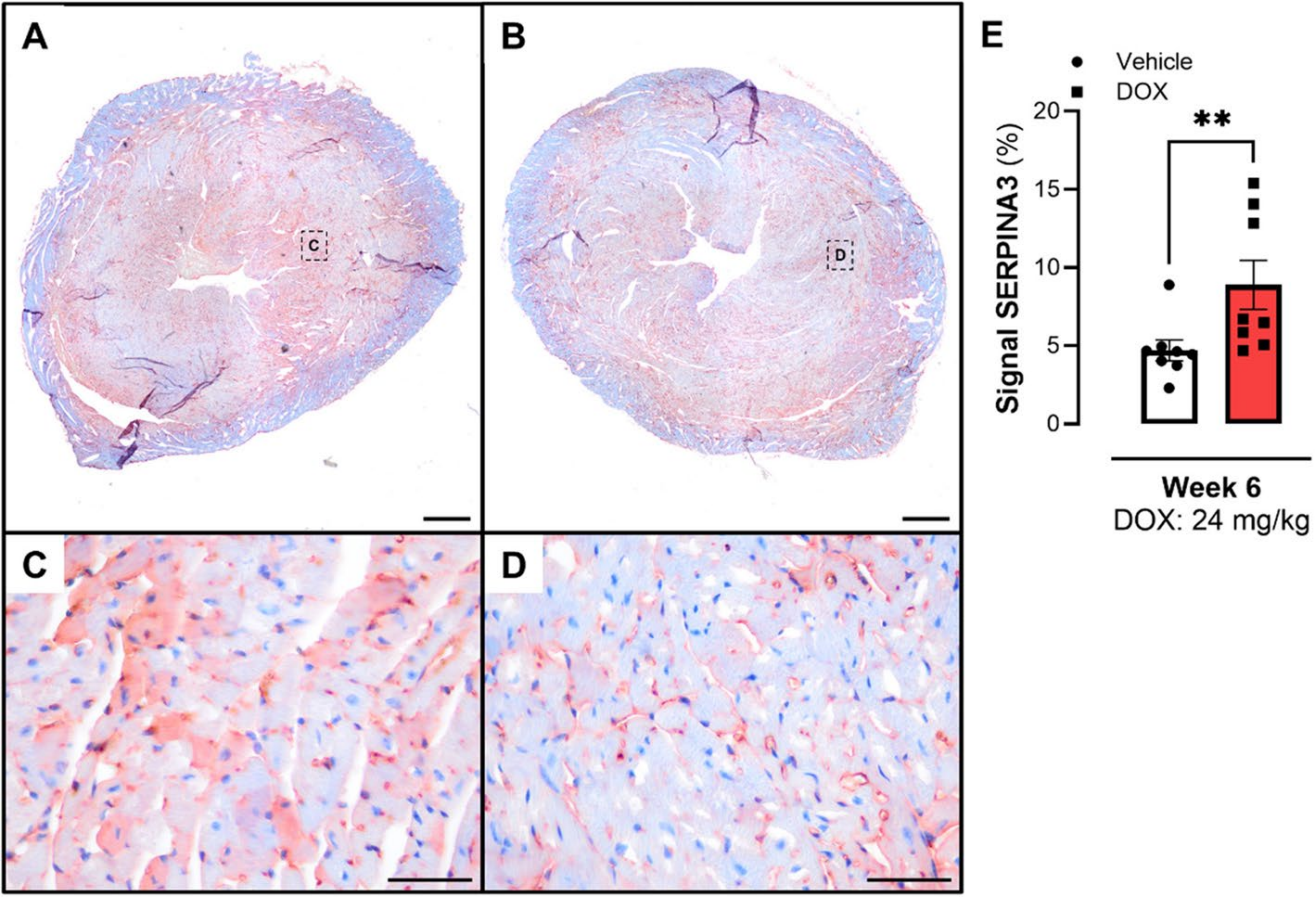
Investigation of SERPINA3 positivity of IHC stained mice cardiac tissue. Positive signal in ECs, cardiomyocites, nuclei, and microvessels. **A** & **C** DOX group. **B** & **D** Vehicle group. **E** SERPINA3 positive signal was higher in the DOX group. Data shows mean ± SEM. **A** & **B** Scale Bar: 1 mm; **C** &**D** Scale Bar: 50 µm. **E** Mann-Whitney test between both groups. ** *p* < 0.01

Cardiac and thoracic aortic tissue from mice after 6 weeks of DOX treatment were stained for SERPINA3. Figure 8 shows cross-section of a representative DOX-treated mouse heart (Fig. 8A & C) and a vehicle mouse heart (Fig. 8B & D). The quantified SERPINA3 signal in cardiac tissue revealed more positivity in the DOX-treated group (Fig. 8E). Positive signal for SERPINA3 was mainly found in the endothelium (i.e., microvessels), but also in cardiomyocytes (Fig. 8C) and co-localized with nuclei (Fig. 8C & D). Besides cardiac tissue SERPINA3 distribution in thoracic aorta was investigated (Supplementary Figure 4A & B). Positive signal was observed in the endothelium and adventitia, as well as in vascular smooth muscle cells.

### SERPINA3 in CTR-CVT patients

**Fig. 9:**
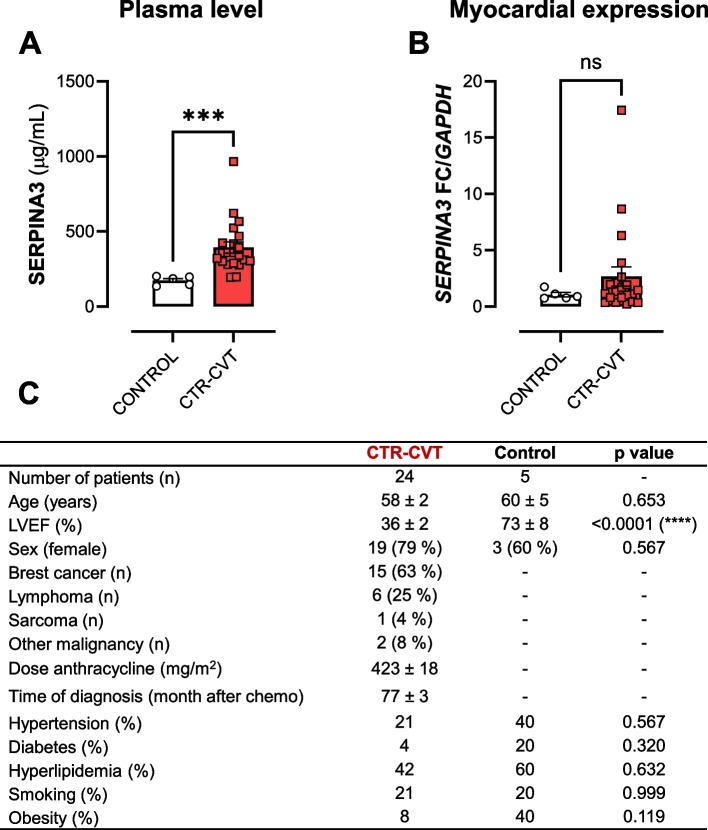
SERPINA3 expression in human LV biopsies and plasma concentration. Two patient cohorts with or without CTR-CVT and same age were investigated. **A** Plasma concentration of SERPINA3. **B** Myocardial gene expression of *SERPINA3*. **C** Table including additional patient-related data. CTR-CVT, Cancer therapy related-cardiovascular toxicity. FC, Fold change. Mann-Whitney test. *** *p* < 0.001

To validate the biomarker potential of SERPINA3, SERPINA3 levels were determined in plasma as well as mRNA expression in myocardial tissue of patients with CTR-CVT compared to controls. Patients with CTR-CVT received on average a cumulative dose of anthracyclines (DOX and equivalents) of 423 ± 12 mg/m^2^. The CTR-CVT group had an average LVEF of 34 ± 2% (Fig. 9C). The control group had an average LVEF of 60 ± 5%. The plasma concentration of circulating SERPINA3 was increased in CTR-CVT patients (Fig. 9A). However, myocardial expression of *SERPINA3* from LV biopsy did not reveal an increase in patients with CTR-CVT compared to control patients (Fig. 9B). Myocardial SERPINA3 expression correlated with circulating SERPINA3 (Pearson = 0.74, *p* = 0.0001) (Supplementary Figure 5). Moreover, myocardial expression of SERPINA3 inversely correlated to LVEF (Pearson = 0.47; *p* = 0.041), while circulating plasma SERPINA3 showed no significant correlation (Pearson = 0.1, *p* = 0,647). Myocardial SERPINA3 did not correlate with dose of anthracycline, while LVEF correlated (Pearson = 0.44; *p* = 0.041).

## Discussion

### DOX-induced CTR-CVT is characterized by both systolic and diastolic dysfunction

The current study focused on diastolic (dys)function as a hallmark of CTR-CVT, based on our previous observation that DOX causes EC dysfunction in an experimental mouse model, even before the onset of systolic dysfunction [11, 12]. The onset of CTR-CVT was confirmed as a reduction in LVEF in the mice. DOX decreased LVEF as of week 2 (cumulative dose of 8 mg/kg), which persisted during further DOX treatment (week 6, cumulative dose of 24 mg/kg of DOX). Yet, the absolute LVEF remained > 50%. Our data are in line with previous data, where similar murine models of chronic DOX-induced CVT with moderately decreased LVEF [12, 20–24]. We were able to demonstrate prolonged τ and decreased dP/dt_min_, which are both hallmarks of diastolic dysfunction by invasive measurements. However, this was only apparent at week 6 and not at week 2 and therefore, after the onset of CTR-CVT. Additionally, we could not detect changes of diastolic function by cardiac ultrasound, as E/E` and E/A ratios were not affected. However, in vivo measurement of diastolic function by ultrasound imaging in mice has a number of technical challenges, such as merged peaks or influences of anaesthesia [25, 26]. Additionally, after 6 weeks we observed increased fluid content in lungs, indicative of pulmonary edema and development of HF [27, 28], although LVEDP was not significantly increased. Possibly the absence of a high LVEDP is related to the anaesthetic sevoflurane, which has vasodilating effects [29].

Taken together, functional and hemodynamic characteristics of our model suggest a mixed systolic and diastolic dysfunction. Diastolic dysfunction is also observed in HFpEF patients and is linked to EC dysfunction as a central mechanism in the underlying pathophysiology [30–32]. As we have previously reported, EC dysfunction occurs in this DOX model [12]. While our data demonstrate that DOX-induced systolic dysfunction precedes diastolic dysfunction in our model, it is important to note that the direct myocardial damage causing systolic dysfunction may also contribute to subsequent diastolic dysfunction [33, 34]. To strengthen the argument linking DOX-induced diastolic dysfunction specifically to EC effects, further investigation is needed to uncouple the direct myocardial dysfunction from the potential impact on EC function within our experimental setup.

Additionally, fibrosis and inflammation are considered central mechanisms in HF patients as well as in CTR-CVT [1, 35, 36]. In our hands, DOX-induced mild fibrosis in the myocardium. Interestingly, it was previously found that DOX (12 mg/kg) triggers fibrosis in mice. This was attributed to mitochondrial damage induced by inflammation [37]. Besides, inflammation in the microvasculature was also found to contribute to sarcomere stiffening [31]. Furthermore, upregulation of cytokines, such as *IL-1*, *IL-6*, *IL-16*, or *TNFα* have been reported in HFpEF patients [38, 39]. Interestingly, we noticed increased mRNA expression of *IL-6* and *TNFα* in murine cardiac tissue. However, the invasive nature of the biopsy procedure hampers clinical applicability. While important, functional markers of CRT-CVT, might not always provide a comprehensive picture of disease or its progression. Molecular biomarkers, on the other hand, offer a deeper insight into underlying processes occurring within the body, often at an earlier stage of the disease when functional changes might not yet be evident. To enlighten molecular changes during the onset and progression of CTR-CVT we performed proteomics on myocardial tissue.

### Myocardial molecular changes identified by proteomics

We identified a consistent increase in myosin heavy chain 7 (MYH7) at both cumulative doses of either 8 mg/kg or 24 mg/kg. Upregulation of MHY7 can be considered a signature of myocardial damage [40, 41], and therefore indicative of DOX-induced CVT. Furthermore, we observed an increased expression of proteins associated with mitochondrial dysfunction (mt-ND1 and mt-CO1) [42, 43]. The mitochondria are considered to be the major target of DOX-induced CVT [44]. This may contribute to mitochondrial dysfunction leading inter alia to an increase in ROS formation [45, 46]. A similar phenomenon is described in HFpEF, resulting in reduced energy production of mitochondria with subsequently increased ROS content [43, 47, 48]. We also found collagen 1 and 6 to be increased after 2, but not 6 weeks of treatment, which could be a sign of early cardiac remodelling [49]. Recently, Abdelgawad et al., applied proteomic profiling to investigate sex-related differences of cumulative 24 mg/kg DOX in C57BL/6N mice. In accordance with our results, they identified an increase of MHY7 as well as a reduction of immunoglobin heavy constant Mu (IGHM) in cardiac tissue of male mice [50]. Additionally, they reported upregulation of markers of cardiac damage such as myosin light chain 7 and natriuretic peptide A. These proteins were not confirmed in another proteomics study using B6C3F1 mice [51]. However, they reported an increase in the CVD marker NOTCH1 and vWF before the onset of myocardial damage (marked as a rise in cardiac troponin) with a cumulative dose of < 12 mg/kg in plasma. We were unable to detect NOTCH1 and vWF in our proteomic analysis. Yuan et al., 2020 found in male Wistar rats treated with DOX (cumulative 18 mg/kg) having CVT, that proteins affecting energy metabolism, oxidative stress regulation and calcium homeostasis were affected [52]. Compared to our analysis, the proteomic profiling in rodent models of DOX-induced CVT shows robust effects on cardiac remodelling, cardiac damage response, and oxidative stress adaptation.

Interestingly, we observed a robust twofold increase in SERPINA3 in cardiac tissue which was confirmed by western blot, qPCR, and IHC. Previously, we also reported upregulation of SERPINA3 in the aorta [12]. Additionally, we could show an increase in circulating SERPINA3 in the mice. *Serpina3* upregulation in the right ventricle of mice treated with DOX (cumulative dose 20 mg/kg) was previously reported as well [21]. In contrast, Abdelgawad, I. Y. et al., 2024, found that SERPINA3K was downregulated in the cardiac tissue of mice treated with cumulative 24 mg/kg of DOX but SERPINA3 was not reported [50]. The discrepancy between their study and the current work may relate to the use of a different strain, although this seems rather unlikely. Alternatively, there might be methodological differences.

Finally, SERPINA1E (alpha 1-antitrypsin), was reduced in both aorta and heart after 2 and 6 weeks of DOX treatment. However, we failed to detect SERPINA1E with either western blot or IHC, and have not continued on this track. The human orthologue of SERPINA1E is SERPINA1, which deficiency is known to be a molecular marker for lung diseases [53]. Colocalization and possible interactions of SERPINA1 and SERPINA3 have been reported in patients with immunological renal disease [54], in brain tissues of Alzheimer’s patients [55], or in patient-derived HLA-positive cervical carcinoma [56]. Nevertheless, SERPINA1 and SERPINA3 may display a complex interplay in CRT-CVT, and further research is warranted.

### SERPINA3, an enigmatic protein with association to CTR-CVT

SERPINA3 is a circulating acute-phase protein and endogenous inhibitor of serine proteases [57]. There is growing evidence that it could be a prognostic marker in cardiovascular disease [58–62]. Yet, its (patho)physiological role is poorly understood, and literature is often contradictory, stressing the need for further research.

An important novelty of the current work is the demonstration of local expression of SERPINA3 in cardiac tissue. Traditionally, literature points to liver as primary source of circulating SERPINA3 [62, 63], yet we examined SERPINA3 distribution in murine cardiac and aortic tissue. SERPINA3 was mainly localized in cardiac and aortic ECs, with lower expression in cardiomyocytes and vascular smooth muscle cells, respectively. Further, we could detect *SERPINA3* in LV biopsies from both CTR-CVT patients as well as controls. Compared to controls, myocardial expression of *SERPINA3* was not increased, whereas plasma levels of SERPINA3 were significantly higher in of CTR-CVT patients compared to controls. This suggests that cardiomyocytes may not be the source of SERPINA3 in DOX-induced CVT. In summary our finding suggests that most SERPINA3 was detected in ECs. Whether ECs produce SERPINA3 or are just a target in the pathophysiological processes involved in CTR-CVT remains unclear. The predominant expression of SERPINA3 in ECs aligns with previous studies, which reported expression of *SERPINA3 *in vitro in human umbilical vein endothelial cell-lines [61, 64].

Our study provides evidence supporting the hypothesis that SERPINA3 may serve as a marker of DOX-induced CVT, as its expression was increased in murine myocardial tissue and plasma after DOX treatment. In addition, we demonstrated increased circulating SERPINA3 in plasma of patients, which is in line with our previous findings [12]. Replication of results across different cohorts reinforces the robustness of the upregulation of SERPINA3 in cardiovascular pathology.

The demographic and clinical characteristics reveal a diverse cohort of patients, primarily comprising individuals treated with anthracycline-based regiments in the setting of breast malignancy, followed by lymphoma, sarcoma, and other malignancies. While we found a relation between dosing of the patients and myocardial SERPINA3 expression, we could not reproduce our previous findings from another patient cohort showing a correlation between LVEF decline and increased circulating SERPINA3 [12]. It is noteworthy that the increase in SERPINA3 in plasma from the CTR-CVT patients aligns with the murine model of DOX-induced CVT. This consistency suggests a potential mechanistic link between chemotherapy exposure and SERPINA3 expression. As such, SERPINA3 could serve as a biomarker or mediator of chemotherapy-associated cardiac dysfunction. Furthermore, the diversity in underlying malignancies and comorbidities among the patients underscores the complexity of cardiotoxicity associated with chemotherapy. Of note, At the same time, we need to take into account, that the plasma concentration in humans was significantly (more than 10,000-fold) higher than in mice. A possible explanation for the difference in circulating SERPINA3 between mice and men could be related to the fact, that mice have 14 isoforms of SERPINA3. The ELISA we used was just specific for the N variant of SERPIAN3 leading to an underestimation of the total SERPINA3 concentration in murine plasma. Further, internal data shows considerable inter-assay variability with values between 10 and 50 ng/mL of SERPINA3, yet with consistent differences between treatment groups. We have previously demonstrated that increased plasma SERPINA3 was associated with a higher risk of mortality in HFrEF patients [62]. While our work shows a clear association of SERPINA3 with CTR-CVT, the exact role of SERPINA3 and mechanisms involved remain unknown. Through inhibition of protease activity related to immune cells, SERPINA3 is considered to dampen inflammation and local tissue damage. In contrast, SERPINA3 also possesses a lesser-explored DNA-binding domain with multifaceted non-canonical actions independent of its serine protease inhibitory functions [65–67]. Accumulation of SERPINA3 in the nuclei has been reported to interfere with DNA polymerase and has been linked to inhibition of cell growth, proliferation, senescence and differentiation [65, 66, 68, 69]. Our IHC images show SERPINA3 positive signal in some nuclei suggesting a possible impact on senescence.

Beyond a possible role in CTR-CVT, SERPINA3 has been investigated in other CV pathologies. SERPINA3 has been shown to be involved in inflammation and cardiac fibrosis [63, 70, 71]. Recent studies reported increased circulating levels of SERPINA3 in patients with dilated cardiomyopathy and acute coronary syndromes [61, 62]. Furthermore, administration of recombinant SERPINA3 has been observed to exacerbate fibrosis in a Coxsackie-induced myocarditis model [71]. Conversely, there are conflicting observations. For instance, while Wagsater’s study on transgenic *Serpina3*-overexpressing mice failed to demonstrate aggravated atherosclerosis when bred with *ApoE*^−/−^ mice [64]*,* there is evidence suggesting that SERPINA3 contributes to cardiovascular disease progression by stimulating inflammatory factors such as *IL-6* and *ICAM-1* in ECs and vascular smooth muscle cells in vitro [59]. On the other hand, inflammatory responses are also known to trigger *SERPINA3* expression in different tissues [57, 61]. In our model, *Il-6* and *Tnfα* were modestly increased in cardiac tissue after DOX treatment, together with an increase in *Serpina3*, which is in line with earlier findings (reviewed elsewhere [57]). Additionally, *Il-1β* has been shown to increase *Serpina3* expression in mice [71]. Alternatively, a recent study demonstrated the pro-inflammatory effects of SERPINA3 in mice, as hepatocyte-specific *Serpina3* knockout was protective against acetaminophen-induced liver inflammation and fibrosis [72]. The contrasting nature of these reports underscores the need for further investigations, including studies utilizing cell-specific *Serpina3*^−/−^ mice, to unravel the complex role of SERPINA3 in cardiac pathologies and CTR-CVT.

## Conclusion

In conclusion, we demonstrated both systolic and diastolic dysfunction in a mouse model of sub-chronic DOX-induced CVT. Proteomics confirmed a number of known pathways in CVT and HF development such as myocardial and mitochondrial damage, as well as an increase in SERPINA3, a protein already linked to cardiovascular disease. We confirmed increased levels of SERPINA3 in mice and CTR-CVT patients. Additionally, IHC analysis revealed that ECs may be the main source of SERPINA3. Collectively, our results show SERPINA3 holds promise as marker of DOX-induced CVT.

### Supplementary Information

Supplementary Material 1: Supplementary Figure 1. Representative H & E staining from cardiac tissue of mice treated either with cumulative 24 mg/kg of DOX, or equivalently of saline. No changes were detected in cellular morphology. A: Image of a vehicle-treated mice. B: Image of Cardiac tissue of a DOX-treated mouse. Scale bar: 500 µm. Supplementary Figure 2. *Enrichr* pathway analysis of differently expressed proteins in myocardial tissue after DOX treatment. A: Pathway analysis of upregulated proteins at 2 and 6 weeks treated mice. B: Pathway analysis of downregulated proteins at 2 and 6 weeks treated mice. Reactome 2022 human database was used. Supplementary Figure 3. Western blots for SERPINA3 and GAPDH of myocardial samples at week 2 and 6. Uncropped blots from Fig. 6. Supplementary Figure 4. SERPINA3 positivity of IHC stained mice thoracic aorta tissue. Positive signal in ECs, vascular smooth muscle cells, A: Thoracic aorta of a DOX mouse. B: Vehicle group: Thoracic aorta of a vehicle-treated mouse. A & B: Scale Bar: 50 µm. Supplementary Figure 5. Correlation plot of myocardial expression and plasma levels of SERPINA3 in patients. A positive correlation between myocardial expression in plasma levels of SERPINA3 could be seen. CTR-CVT: cancer therapy-related-cardiovascular toxicity.

Supplementary Material 2.

## Data Availability

The datasets used and/or analyzed during the current study are available from the corresponding author on reasonable request

## Abbreviations

CTR-CVT: Cancer therapy-related cardiovascular toxicity
DOX: Doxorubicin
EC: Endothelial cells
FS: Fractional shortening
GLS: Global longitudinal strain
HF: Heart failure
HFpEF: Heart failure with preserved ejection fraction
HFrEF: Heart failure with reduced ejection fraction
IHC: Immunohistochemistry
LV: Left ventricle
LVEDP: Left ventricular end-diastolic pressure
LVEF: Left ventricular ejection fraction
LVP: Left ventricular pressure
qPCR: Quantitative real-time polymerase chain reaction
