# Supplementary material for: Characterization of systolic and diastolic function, alongside proteomic profiling, in doxorubicin-induced cardiovascular toxicity in mice
Source: Cardiooncology. 2024 Jun 22;10:40. doi: 10.1186/s40959-024-00241-1 (PMC11193203; doi:10.1186/s40959-024-00241-1)
Supplement: Supplementary file 1 — Supplementary Material 1: Supplementary Figure 1. Representative H & E staining from cardiac tissue of mice treated either with cumulative 24 mg/kg of DOX, or equivalently of saline. No changes were detected in cellular morphology. A: Image of a vehicle-treated mice. B: Image of Cardiac tissue of a DOX-treated mouse. Scale bar: 500 µm. Supplementary Figure 2. Enrichr pathway analysis of differently expressed proteins in myocardial tissue after DOX treatment. A: Pathway analysis of upregulated proteins at 2 and 6 weeks treated mice. B: Pathway analysis of downregulated proteins at 2 and 6 weeks treated mice. Reactome 2022 human database was used. Supplementary Figure 3. Western blots for SERPINA3 and GAPDH of myocardial samples at week 2 and 6. Uncropped blots from Fig. 6. Supplementary Figure 4. SERPINA3 positivity of IHC stained mice thoracic aorta tissue. Positive signal in ECs, vascular smooth muscle cells, A: Thoracic aorta of a DOX mouse. B: Vehicle group: Thoracic aorta of a vehicle-treated mouse. A & B: Scale Bar: 50 µm. Supplementary Figure 5. Correlation plot of myocardial expression and plasma levels of SERPINA3 in patients. A positive correlation between myocardial expression in plasma levels of SERPINA3 could be seen. CTR-CVT: cancer therapy-related-cardiovascular toxicity. [file 40959_2024_241_MOESM1_ESM.docx]

# **Supplementary Figures**


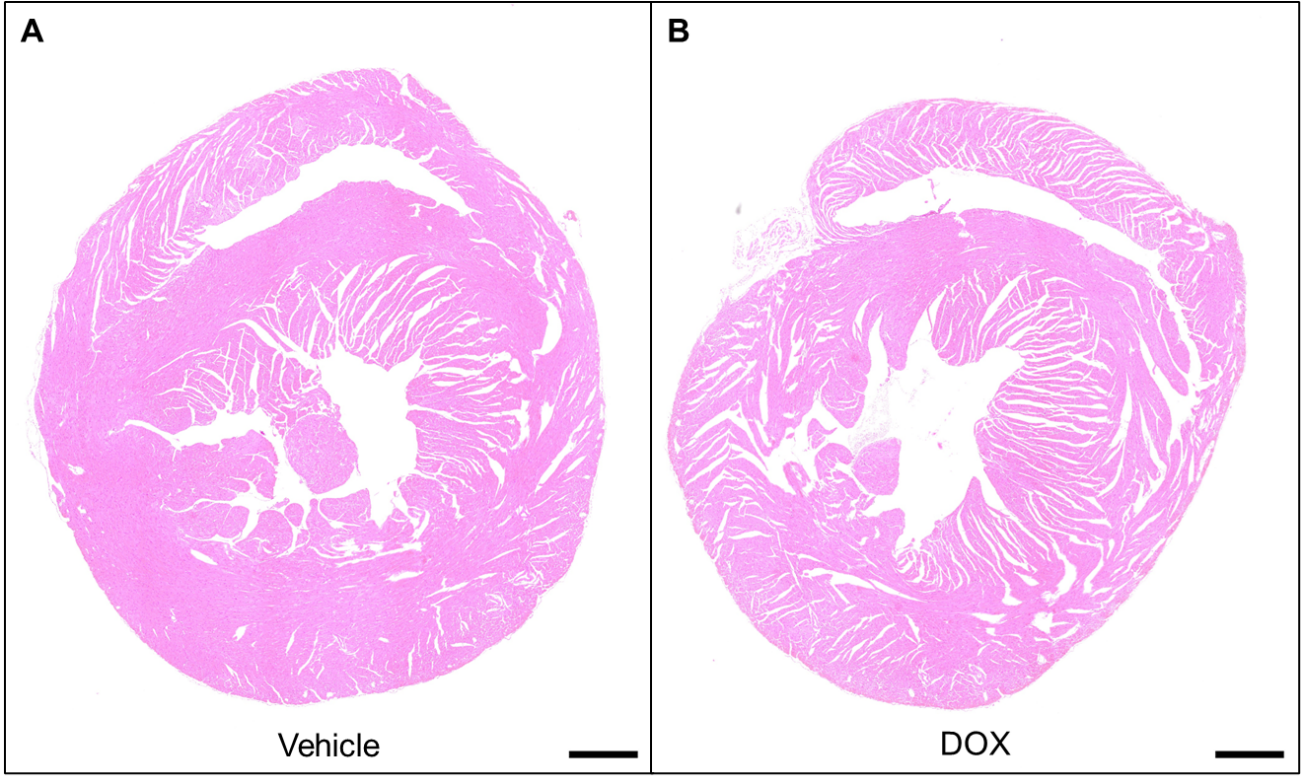


**Supplementary Figure 1: Representative H & E staining from cardiac tissue of mice treated either with cumulative 24 mg/kg of DOX, or equivalently of saline**. No changes were detected in cellular morphology. **A:** Image of a vehicle-treated mice. **B:** Image of Cardiac tissue of a DOX-treated mouse. Scale bar: 500 µm.

**Supplementary Figure 2:** **Enrichr pathway analysis of differently expressed proteins in myocardial tissue after DOX treatment. A:** Pathway analysis of upregulated proteins at 2 and 6 weeks treated mice. **B:** Pathway analysis of downregulated proteins at 2 and 6 weeks treated mice. Reactome 2022 human database was used.


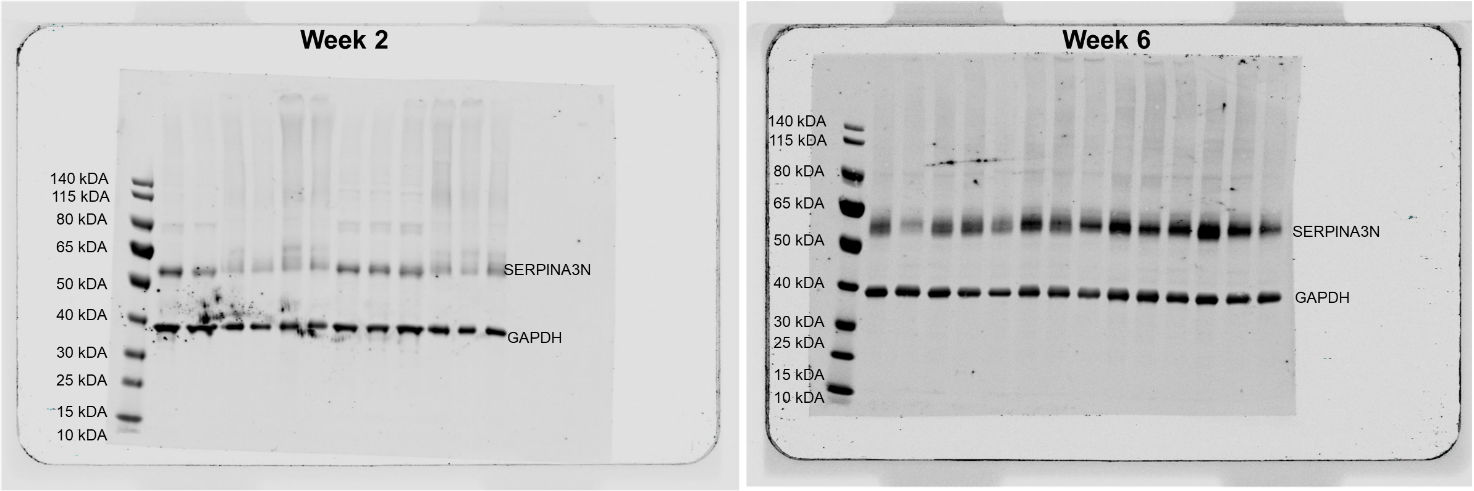


**Supplementary Figure 3:** **Western blots for SERPINA3 and GAPDH of myocardial samples at week 2 and 6.** Uncropped blots from Figure 6.

**
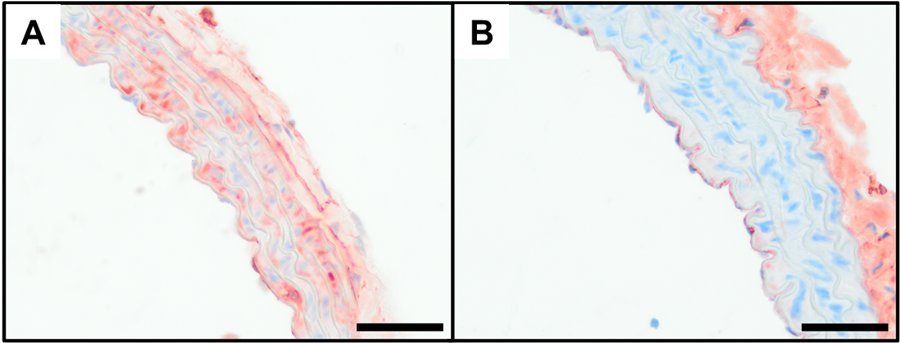
**

**Supplementary Figure 4:** **SERPINA3 positivity of IHC stained mice thoracic aorta tissue.** Positive signal in ECs, vascular smooth muscle cells, **A:** Thoracic aorta of a DOX mouse. **B:** vehicle group: Thoracic aorta of a vehicle-treated mouse. A & B: Scale Bar: 50 µm

**Supplementary Figure 5:** **Correlation plot of myocardial expression and plasma levels of SERPINA3 in patients.** A positive correlation between myocardial expression in plasma levels of SERPINA3 could be seen. CTR-CVT: cancer therapy-related-cardiovascular toxicity
